# Identification of candidate intergenic risk loci in autism spectrum disorder

**DOI:** 10.1186/1471-2164-14-499

**Published:** 2013-07-24

**Authors:** Susan Walker, Stephen W Scherer

**Affiliations:** 1 Program in Genetics and Genome Biology, The Centre for Applied Genomics, The Hospital for Sick Children, Toronto, Ontario M5G 1L7, Canada; 2 McLaughlin Centre, University of Toronto, Toronto, Ontario M5S 1A1, Canada

**Keywords:** Autism spectrum disorder, Copy number variation, Non-coding DNA

## Abstract

**Background:**

Copy number variations (CNVs) and DNA sequence alterations affecting specific neuronal genes are established risk factors for Autism Spectrum Disorder (ASD). In what is largely considered a genetic condition, so far, these mutations account for ~20% of individuals having an ASD diagnosis. However, non-coding genomic sequence also contains functional elements introducing additional disease risk loci for investigation.

**Results:**

We have performed genome-wide analyses and identified rare inherited CNVs affecting non-genic intervals in 41 of 1491 (3%) of ASD cases examined. Examples of such intergenic CNV regions include 16q21 and 2p16.3 near known ASD risk genes *CDH8* and *NRXN1* respectively, as well as novel loci contiguous with *ZHX2*, *MOCS1*, *LRRC4C*, *SEMA3C,* and other genes.

**Conclusions:**

Rare variants in intergenic regions may implicate new risk loci and genes in ASD and also present useful data for comparison with coming whole genome sequence datasets.

## Background

Newer genomic technologies like high-resolution microarrays and next generation exome sequencing have enabled the identification of many clinically relevant genetic variants for both Mendelian and complex disorders. Yet for many conditions the identified genes account for only a proportion of heritability. This observation coupled with the recognition of the functional relevance of non-genic regions [1] target these genomic segments as candidates for investigation for a role in disease.

ASD encompasses a range of neurodevelopmental disorders characterised by social impairment, communication difficulties and restricted, repetitive behavioural patterns. ASD, which is clinically and genetically heterogeneous, demonstrates high heritability, familial clustering and ~4:1 male to female bias. While there has been progress identifying risk genes, most are still unknown [2]. Analyses of rare (<1% population frequency) CNVs, insertions and deletions (indels) and point mutations have most convincingly identified synaptic genes such as members of the Neuroligin (*NLGN3*, *NLGN4)*[3], Neurexin (*NRXN1 *[4], *NRXN2 *[5]*, NRXN3 *[6]), SHANK (*SHANK1 *[7], *SHANK2 *[8], *SHANK3 *[9]) families and Gephyrin [10] as highly-penetrant risk loci [2]. ASD subjects with multiple genetic risk factors for ASD and associated medical conditions are also known [11]. In addition, there are a few examples of mutations in ASD cases identified in non-genic segments of DNA [12] and non-coding RNAs [13]. Similar findings are even better documented in studies of intellectual disability [14,15], which is observed in ~40% of cases of ASD. Focusing on the intergenic intervals of the genome, we performed a systematic genome-wide investigation to identify rare CNVs enriched in cases compared with controls [16] to identify known and novel ASD susceptibility loci.

## Methods

A collection of 1491 unrelated ASD cases were genotyped using either the Illumina 1M (993) or the Affymetrix SNP 6.0 platforms (498). The ASD subjects, all diagnosed using gold-standard instruments including Autism Diagnostic Interview and Autism Diagnostic Observation Schedule, are described elsewhere [16,17]. Informed written consent was obtained from all participants, as approved by the Research Ethics Boards at The Hospital for Sick Children and McMaster University. For controls, 1287 samples from the SAGE cohort were genotyped on with the Illumina 1M and 1234 samples from the Ottawa Heart Institute (OHI) and 1123 from the POPGEN collections were genotyped on the Affymetrix SNP 6.0. CNV discovery was performed using previously described pipelines [16-18]. Three CNV detection tools were used for each platform (Birdsuite, iPattern and Genotyping Console for Affymetrix 6.0 and iPattern, QuantiSNP & PennCNV for Illumina 1 M). A subset of CNVs in both cases and controls were considered rare if they were present in <1% of the overall dataset and these were further analysed if they failed to intersect or fall within a known gene (according to the NCBI Reference Sequence (RefSeq), August 2011). Rare genic CNVs identified from these data have been reported previously and from these data approximately 10% of cases carry a *de novo* or rare inherited CNV thought to contribute to ASD in that individual [16,17,19,20]. All CNVs discussed were validated where DNA was available using independent laboratory methods such as long range or quantitative PCR and the mode of inheritance determined (Additional files 1 and 2).

## Results and discussion

Microarray data from a cohort of 1491 unrelated ASD probands were analysed for rare copy number variants as described previously [16,17] and CNVs falling outside of known coding sequence were identified. A total of 212 non-coding genomic regions were determined as harboring overlapping CNVs in two or more unrelated ASD cases that were absent in control samples. Each region was examined for plausible biological function by comparison with multiple databases. Data was collated for evidence of expressed sequences from mRNA or EST data at GenBank or evolutionary conservation as well as functional predictions from the VISTA enhancer browser (http://enhancer.lbl.gov/) and Rfam (http://rfam.sanger.ac.uk/). The Database of Genomic Variants (http://dgvbeta.tcag.ca/dgv/app/home) was used to eliminate additional regions as non-ASD specific CNVs and regions with >80% masked as repetitive sequences were removed. Loci were also prioritised as being of potential clinical significance in ASD due to proximity to genes considered known or candidate ASD risk genes [17].

**Figure 1:**
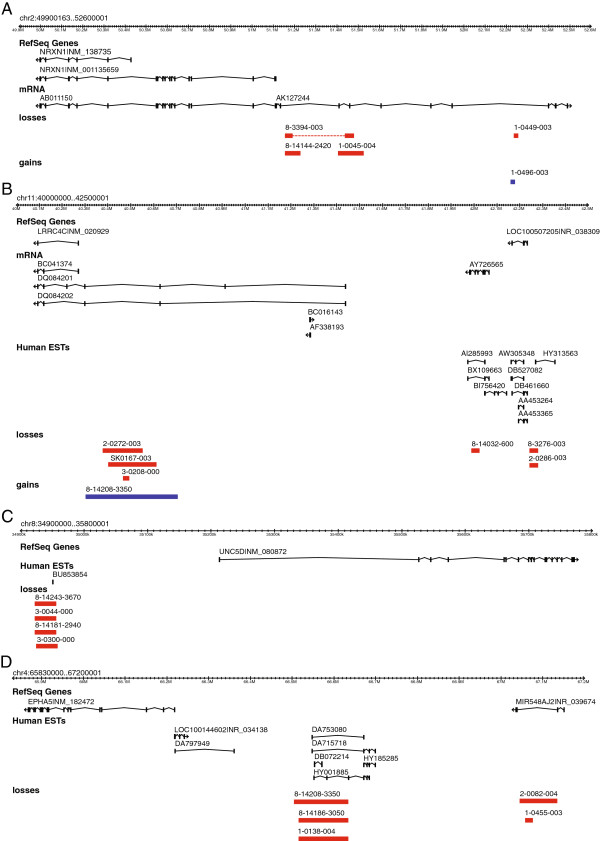
**Genome browser views of ASD specific CNVs at A) 2p16.3 B) 11p12 C) 8p12 and D) 4q13.1.** In each case, representative isoforms of known RefSeq genes, mRNA and/or Expressed Sequence Tags are shown. Deletions and duplications are represented by red and blue bars, respectively. In Figure 1A) a dashed line indicates a diploid region located between two adjacent deletions in the same individual. Additional browser views from other loci shown in Table 1 are included in Additional file 1 A-J. In all cases where parental DNA was available, the CNVs shown were found to be inherited. Additional case SK0167-003 found in Marshall *et al.*[19].

Fifteen intergenic regions emerged as plausible candidate ASD risk loci and in all instances the defining CNV events were inherited. In one of these regions, an additional case (SK0167-003) was found with an overlapping CNV described by Marshall *et al.* (2008) [19] (Table 1, Figure 1 and Additional files 1 and 2). In 14 of 15, the intergenic interval identified has not been described before and in three regions the CNV neighboured a known ASD gene, namely, *CDH8* [21], *C3orf58* [22] and *NRXN1* [4]. In the case of the *NRXN1* gene, upstream CNVs found in five individuals impact the same mRNA (AK127244) reported elsewhere with a CNV in a family with ASD (Table 1, Figure 1A) [23]. Examples of other intergenic CNVs identified highlight regions at 8q24.12 upstream of *ZHX2*, 6p21.2 upstream of *MOCS1*, 11p12 upstream of *LRRC4C* (Figure 1B) and 7q21.11 upstream of *SEMA3C*, as putative novel ASD rearrangements. In one case (8-14208-3350), deletions were identified at three separate loci; 4q13.1 upstream of *EPHA5*, 11p14.3 upstream of *LUZP2* and 11p12 upstream of *LRRC4C* and another case (3-0496-003) carried a 46, XXY sex chromosome imbalance. Other CNVs found in these 41 cases are shown in Additional file 3 and any or all of these may be contributing to the genetic load for ASD [11,17]. Interestingly, all the CNVs identified through our analysis are inherited events. The significance of this observation is still to be determined but suggests incomplete and/or variable penetrance of phenotype, which is something often observed in ASD [6,7,17].

**Table 1:** ASD specific CNVs in intergenic regions

**Locus**	**Gene**	**Sample**	**CNV**	**Start**	**End**	**Size**	**Furthest distance from gene**	**Bin**
2p16.3	*NRXN1* AK127244 mRNA	1-0045-004	loss	51405882	51524684	118802	1124	ii
		8-3394-003	loss	51439897	51479683	39786		
		8-3394-003	loss	51157414	51189362	31948		
		8-14144-2420	loss	51157414	51225851	68437		
		1-0496-003	gain	52220120	52238172	18052		
		1-0449-003	loss	52237072	52253660	16588		
3p22.3	*ARPP21*	2-1213-003	loss	34984049	35102773	118724	563	ii
		3-0100-000	gain	35086691	35094736	8045		
3q24	*C3orf58 ZIC1, ZIC4*	1-0007-003	loss	146168760	146934953	766193	1383 1955, 1979	i
		8-3093-004	loss	146575437	146631141	55704		
4q13.1	*EPHA5*	8-14208-3350	loss	66505324	66633530	128206	840	i
		8-14186-3050	loss	66515708	66633530	117822		
		1-0138-004	loss	66515708	66633530	117822		
		2-0082-004	loss	67045815	67134170	88355		
		1-0455-003	loss	67058506	67075558	17052		
6p21.2	*MOCS1*	3-0139-000	gain	40021898	40078515	56617	168	i or ii
		2-0139-003	gain	40023327	40062155	38828		
		1-0381-003	loss	40174188	40209324	35136		
		2-1368-003	loss	40174188	40210694	36506		
7q21.11	*SEMA3C*	8-6258-03	loss	80431202	80512022	80820	96	i
		1-0345-005	loss	80482597	80517630	35033		
8p12	*UNC5D NRG1*	8-14243-3670	loss	34923482	34956067	32585	256 2183	i
		3-0044-000	loss	34923482	34956067	32585		
		3-0300-000	loss	34925149	34957854	32705		
		8-14181-2940	loss	34923482	34956067	32585		
8q24.13	*ZHX2*	8-3317-003	gain	123572785	123625681	52896	237	i or ii
		3-0186-000	loss	123583028	123639417	56389		
9q33.1	*ASTN2*	8-3055-004	loss	119254497	119374796	120299	98	i
		3-0115-000	loss	119314967	119319559	4592		
9q34.2	*OLFM1 RXRA*	2-1272-003	gain	136479329	136604233	124904	508 8	i
		2-1189-003	gain	136480334	136598491	118157		
11p14.3	*LUZP2*	8-14175-2820	loss	24177612	24316053	138441	160	i or ii
		8-14059-1020	loss	24262511	24303132	40621		
		8-14208-3350	loss	24262511	24303132	40621		
11p12	*LRRC4C*	8-14208-3350	gain	40304880	40703298	398418	196	iii
		2-0272-003	loss	40379668	40550356	170688		
		SK0167-003	loss	40417554	40610400	192846		
		3-0208-000	loss	40468058	40492541	24483		
11p12	*LRRC4C*	8-14032-600	loss	41990280	42021250	30970	1738	i or ii
		8-3276-003	loss	42243624	42279094	35470		
		2-0286-003	loss	42243624	42279094	35470		
11q13.2	*MRGPRD*	4-0023-003	loss	68486121	68493638	7517	10	i
		2-1075-003	loss	68486121	68500238	14117		
16q21	*CDH8*	8-14251-3750	loss	61650435	61787984	137549	1030	i or ii
		2-1175-003	loss	61658675	61755232	96557		

Location and size of all CNVs discovered are listed with the proposed associated candidate gene. Bin denotes possible mechanism of action by i) altering sequence elements required for regulating expression of neighboring genes ii) affecting undiscovered genes or non-coding RNAs iii) disrupting uncharacterised isoforms of adjacent genes. Genome browser views of all loci are shown in Figure 1 and Additional file 1. All pedigrees are shown in Additional file 2. Additional sample SK0167-003 identified in reference [19].

The mechanism of action of these rare CNVs in the pathogenesis of ASD could be (i) through altering the necessary copy number or positional context of key DNA sequence elements required for regulating the proper expression of nearby genes [1], (ii) affecting still undiscovered genes or non-coding RNAs residing in the CNV regions and (iii) disrupting uncharacterized isoforms of the adjacent annotated genes. In the first scenario, we find CNVs both upstream (e.g. *UNC5D* (Figure 1C), *MOCS1*, *ASTN2*, *SEMA3C*, *ZHX2*, *LUZP2*, *CDH8*) and down-stream (*C3orf58*, *RXRA*, *MRGPRD*) of known ASD risk genes and putative novel loci. For at least three regions (4q13.1, 6p21.2 and 11p12 (shown in Figure 1D, Additional file 1C and Figure 1B respectively)), our CNV mapping data in fact identify two distinct clusters of CNVs at the same locus, all overlapping spliced ESTs and thus with a possible regulatory role. Secondly, three independent CNV deletions interrupting a collection of spliced expressed sequenced tags approximately 330 kb proximal to *EPHA5* highlight a potentially newly discovered ASD risk gene (Figure 1D). Finally, longer isoforms of *LRRC4C* likely exist given the discovery of mRNAs DQ084201 and DQ084202. There are, of course, other functional DNA elements or modifications that need to be considered [24] as the mapping resolution increases.

## Conclusions

Given the challenges faced in interpreting the clinical significance of multitudes of genetic variants found in for example, whole genome sequencing [25], accruing evidence across multiple studies will advocate loci outside of known genes or other regulatory elements for further study, particularly for rare variants. In this light, these data provide a useful resource for comparison as new data sets of both CNVs and nucleotide-level variants become available to help fine-map additional discover new ASD risk loci. This general research strategy can also be applied to other disease gene studies.

## Competing interests

The authors declare that they have no competing interests.

## Authors’ contributions

SW and SWS conceived the project and wrote the manuscript. SW designed the analysis, interpreted the data and conducted laboratory validation experiments. Both authors read and approved the final manuscript.

## Supplementary Material

Additional file 1 Genome Browser views of loci with ASD specific CNVs. Click here for file

Additional file 2 **Pedigree structure for all families listed in Table** 1**.** Click here for file

Additional file 3 Table of all rare CNVs detected in the individuals described herein. Click here for file
