# Supplementary material for: Identification of candidate intergenic risk loci in autism spectrum disorder
Source: BMC Genomics. 2013 Jul 24;14:499. doi: 10.1186/1471-2164-14-499 (PMC3734099; doi:10.1186/1471-2164-14-499)
Supplement: Additional file 1 — Genome Browser views of loci with ASD specific CNVs. [file 1471-2164-14-499-S1.pdf]

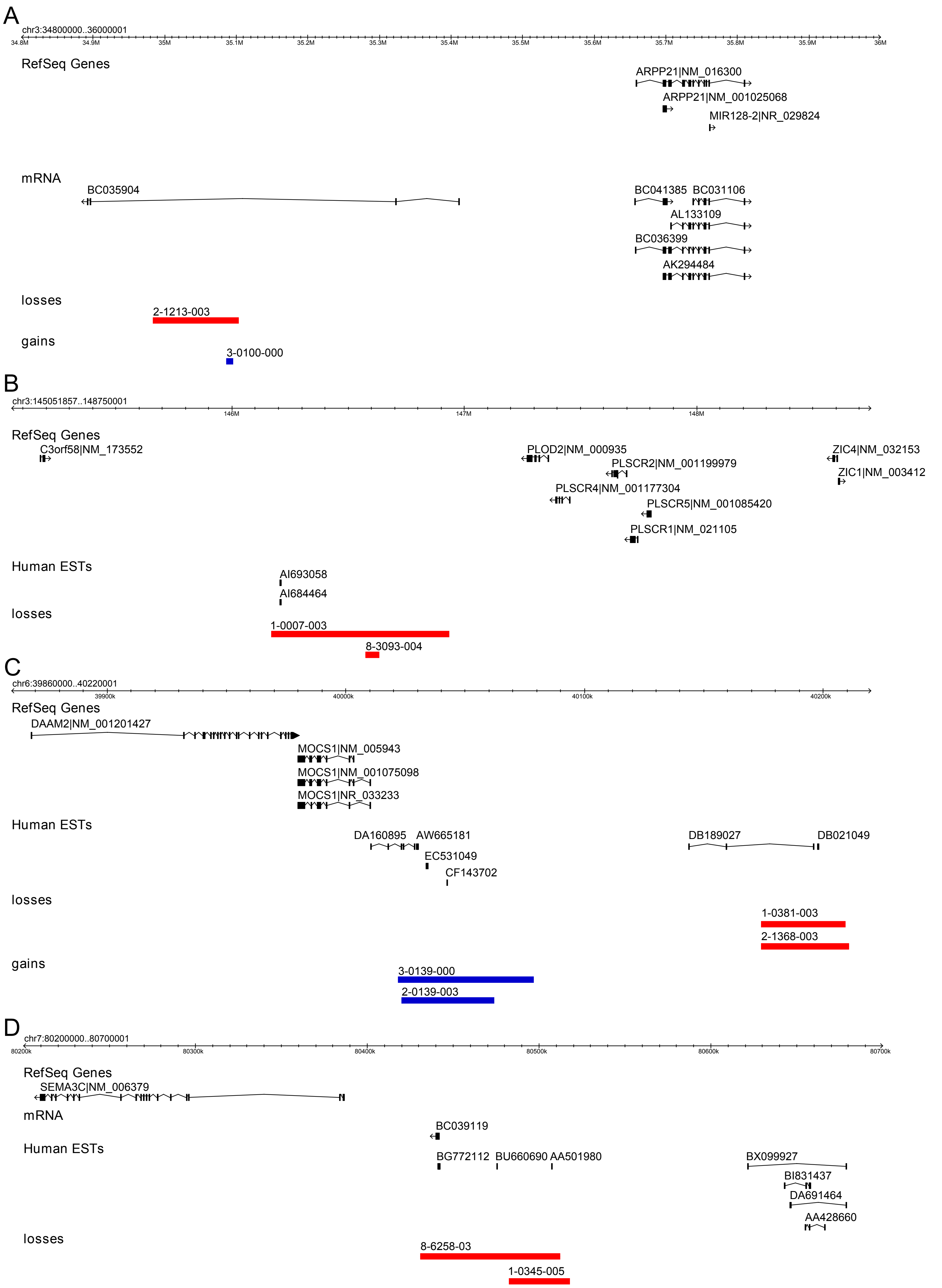
A
chr3:34800000..36000001
34.8M
34.9M
35M
35.1M
35.2M
35.3M
35.4M
35.5M
35.6M
35.7M
35.8M
35.9M
36M
RefSeq Genes
ARPP21|NM_016300
ARPP21|NM_001025068
MIR128-2|NR_029824
mRNA
BC035904
BC041385
BC031106
AL133109
BC036399
AK294484
losses
2-1213-003
gains
3-0100-000
B
chr3:145051857..148750001
146M
147M
148M
RefSeq Genes
C3orf58|NM_173552
PLOD2|NM_000935
PLSCR2|NM_001199979
PLSCR4|NM_001177304
PLSCR5|NM_001085420
PLSCR1|NM_021105
ZIC4|NM_032153
ZIC1|NM_003412
Human ESTs
AI693058
AI684464
losses
1-0007-003
8-3093-004
C
chr6:39860000..40220001
39900k
40000k
40100k
40200k
RefSeq Genes
DAAM2|NM_001201427
MOCS1|NM_005943
MOCS1|NM_001075098
MOCS1|NR_033233
Human ESTs
DA160895
AW665181
EC531049
CF143702
DB189027
DB021049
losses
1-0381-003
2-1368-003
gains
3-0139-000
2-0139-003
D
chr7:80200000..80700001
80200k
80300k
80400k
80500k
80600k
80700k
RefSeq Genes
SEMA3C|NM_006379
mRNA
BC039119
Human ESTs
BG772112
BU660690
AA501980
BX099927
BI831437
DA691464
AA428660
losses
8-6258-03
1-0345-005

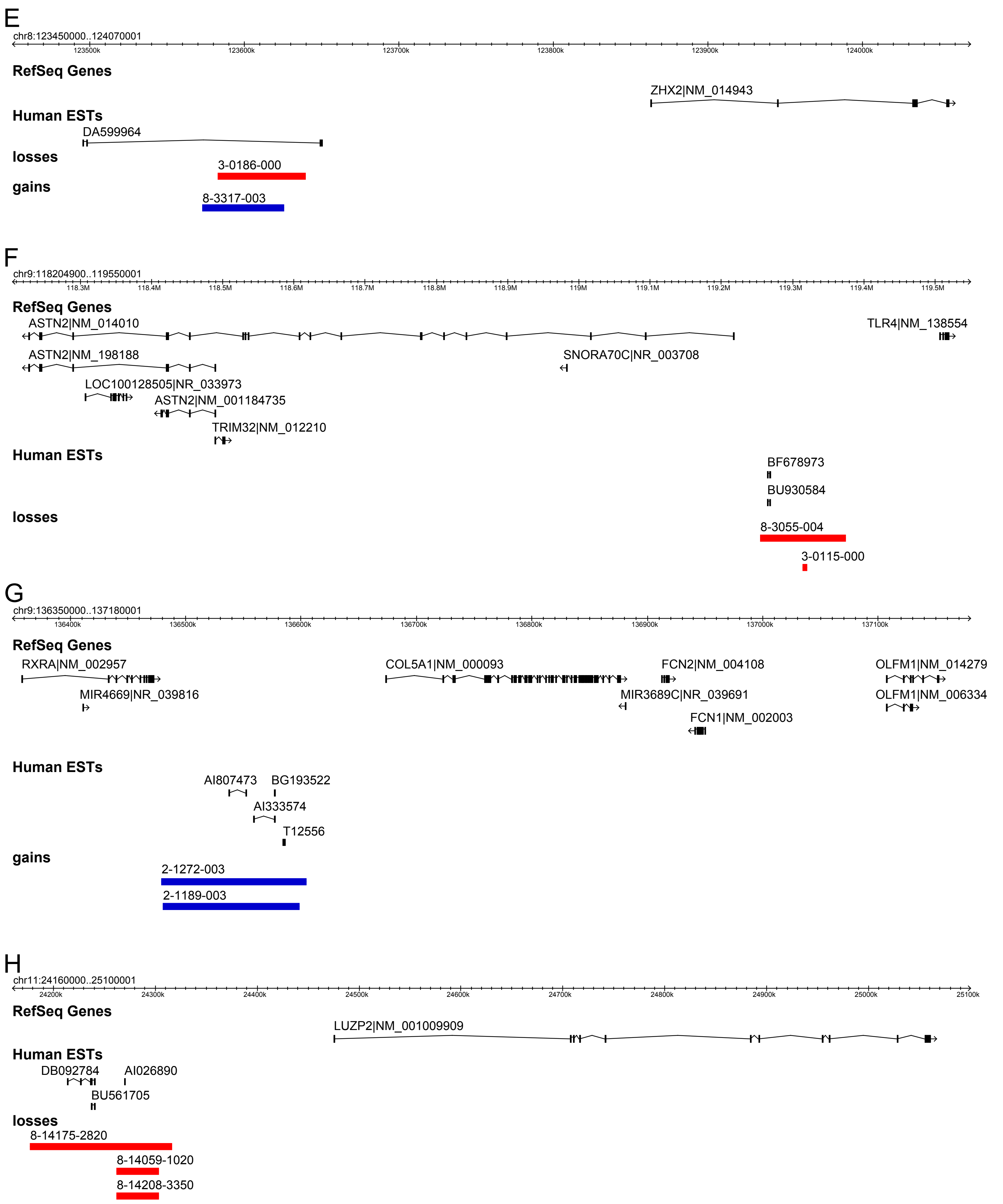
E
chr8:123450000..124070001
123500k
123600k
123700k
123800k
123900k
124000k
RefSeq Genes
ZHX2|NM_014943
Human ESTs
DA599964
losses
3-0186-000
gains
8-3317-003
F
chr9:118204900..119550001
118.3M
118.4M
118.5M
118.6M
118.7M
118.8M
118.9M
119M
119.1M
119.2M
119.3M
119.4M
119.5M
RefSeq Genes
ASTN2|NM_014010
TLR4|NM_138554
ASTN2|NM_198188
SNORA70C|NR_003708
LOC100128505|NR_033973
ASTN2|NM_001184735
TRIM32|NM_012210
Human ESTs
BF678973
BU930584
losses
8-3055-004
3-0115-000
G
chr9:136350000..137180001
136400k
136500k
136600k
136700k
136800k
136900k
137000k
137100k
RefSeq Genes
RXRA|NM_002957
COL5A1|NM_000093
FCN2|NM_004108
OLFM1|NM_014279
MIR4669|NR_039816
MIR3689C|NR_039691
OLFM1|NM_006334
FCN1|NM_002003
Human ESTs
AI807473
BG193522
AI333574
T12556
gains
2-1272-003
2-1189-003
H
chr11:24160000..25100001
24200k
24300k
24400k
24500k
24600k
24700k
24800k
24900k
25000k
25100k
RefSeq Genes
LUZP2|NM_001009909
Human ESTs
DB092784
AI026890
BU561705
losses
8-14175-2820
8-14059-1020
8-14208-3350

I

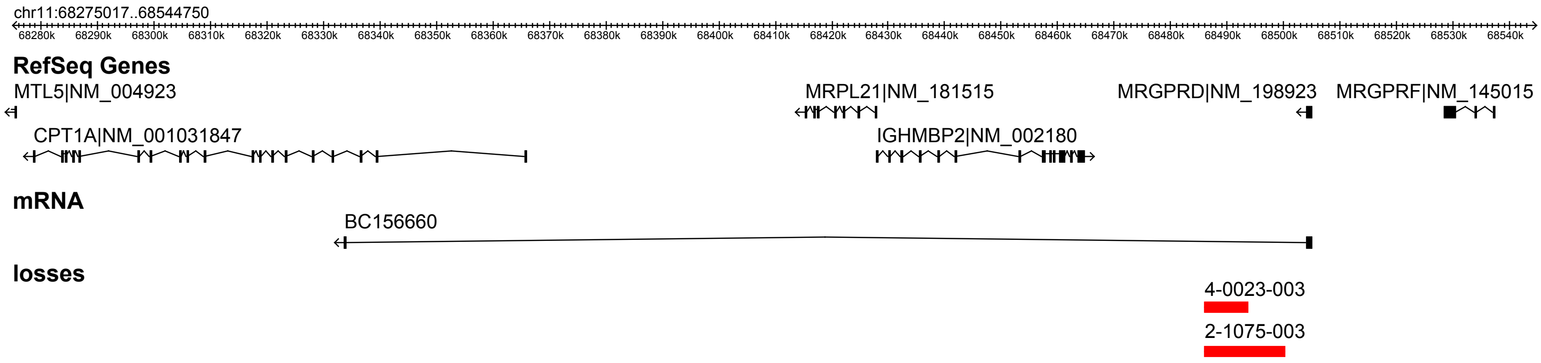

J

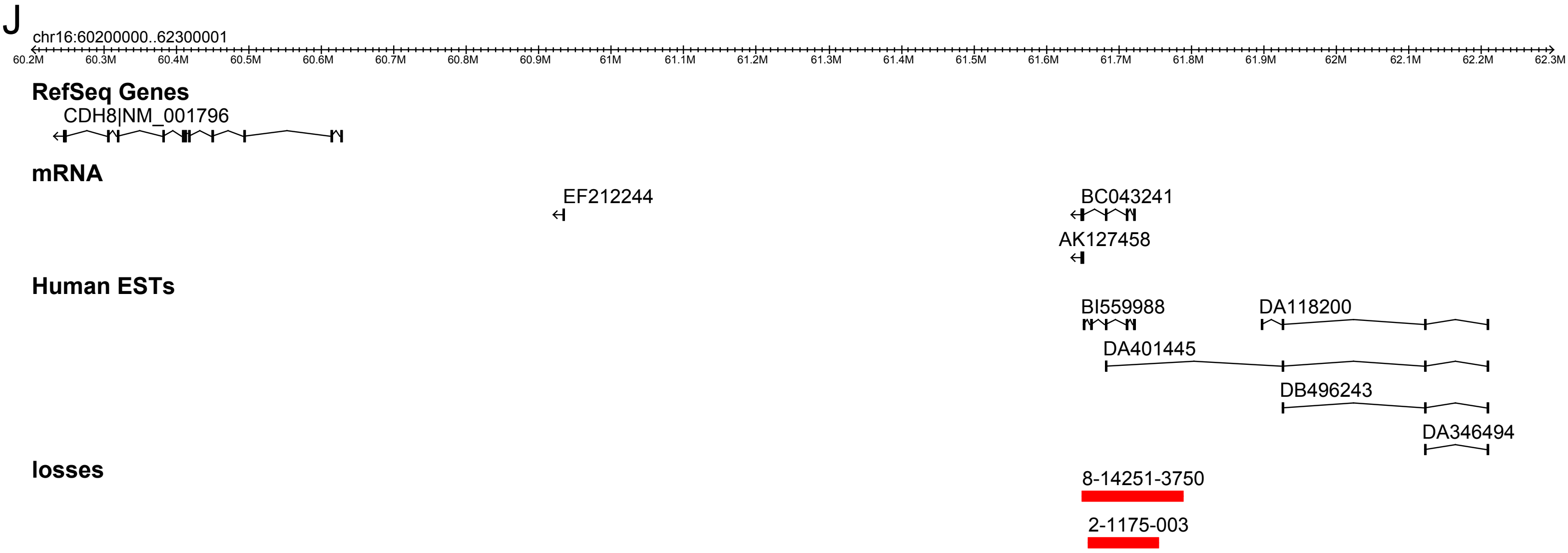

Additional file 1: Genome Browser views of loci with ASD specific CNVs at A) 3p22.3 B) 3q24 C) 6p21.2 D) 7q21.11 E) 8q24.13 F) 9q33.1 G) 9q34.2 H) 11p14.3 I) 11q13.2 J) 16q21.

Representative isoforms of known RefSeq genes, mRNA and/or expressed sequence tags are shown. Deletions and duplications indentified in this study are shown by red and blue bars respectively.
