# Supplementary material for: Identification of candidate intergenic risk loci in autism spectrum disorder
Source: BMC Genomics. 2013 Jul 24;14:499. doi: 10.1186/1471-2164-14-499 (PMC3734099; doi:10.1186/1471-2164-14-499)
Supplement: Additional file 2 — Pedigree structure for all families listed in Table 1. [file 1471-2164-14-499-S2.pdf]

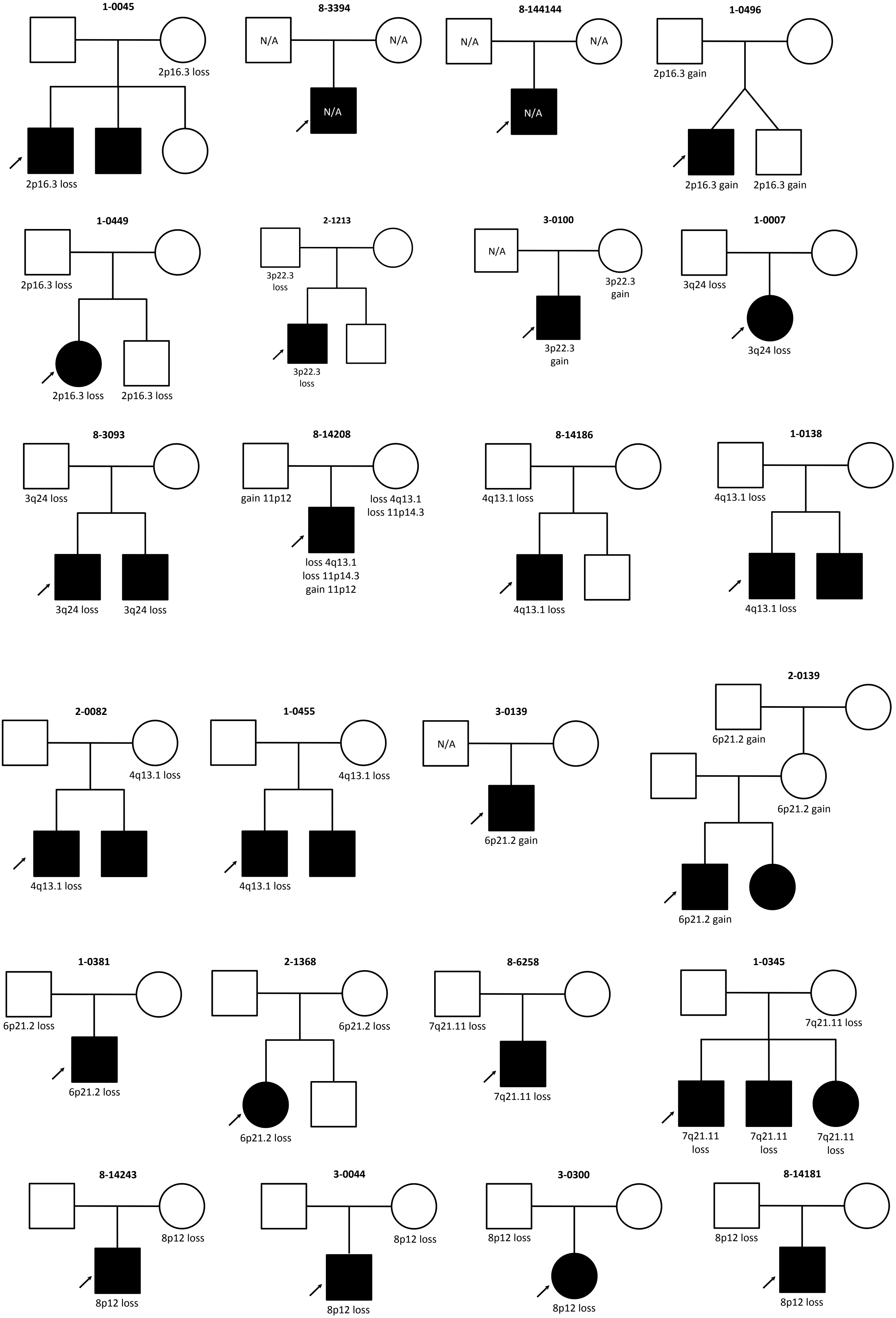
1-0045
2p16.3 loss
2p16.3 loss
8-3394
N/A
N/A
N/A
8-144144
N/A
N/A
N/A
1-0496
2p16.3 gain
2p16.3 gain
2p16.3 gain
1-0449
2p16.3 loss
2p16.3 loss
2p16.3 loss
2-1213
3p22.3 loss
3p22.3 loss
3-0100
N/A
3p22.3 gain
3p22.3 gain
1-0007
3q24 loss
3q24 loss
8-3093
3q24 loss
3q24 loss
3q24 loss
8-14208
gain 11p12
loss 4q13.1
loss 11p14.3
loss 4q13.1
loss 11p14.3
gain 11p12
8-14186
4q13.1 loss
4q13.1 loss
1-0138
4q13.1 loss
4q13.1 loss
2-0082
4q13.1 loss
4q13.1 loss
1-0455
4q13.1 loss
4q13.1 loss
3-0139
N/A
6p21.2 gain
2-0139
6p21.2 gain
6p21.2 gain
6p21.2 gain
1-0381
6p21.2 loss
6p21.2 loss
2-1368
6p21.2 loss
6p21.2 loss
8-6258
7q21.11 loss
7q21.11 loss
1-0345
7q21.11 loss
7q21.11 loss
7q21.11 loss
7q21.11 loss
8-14243
8p12 loss
8p12 loss
3-0044
8p12 loss
8p12 loss
3-0300
8p12 loss
8p12 loss
8-14181
8p12 loss
8p12 loss

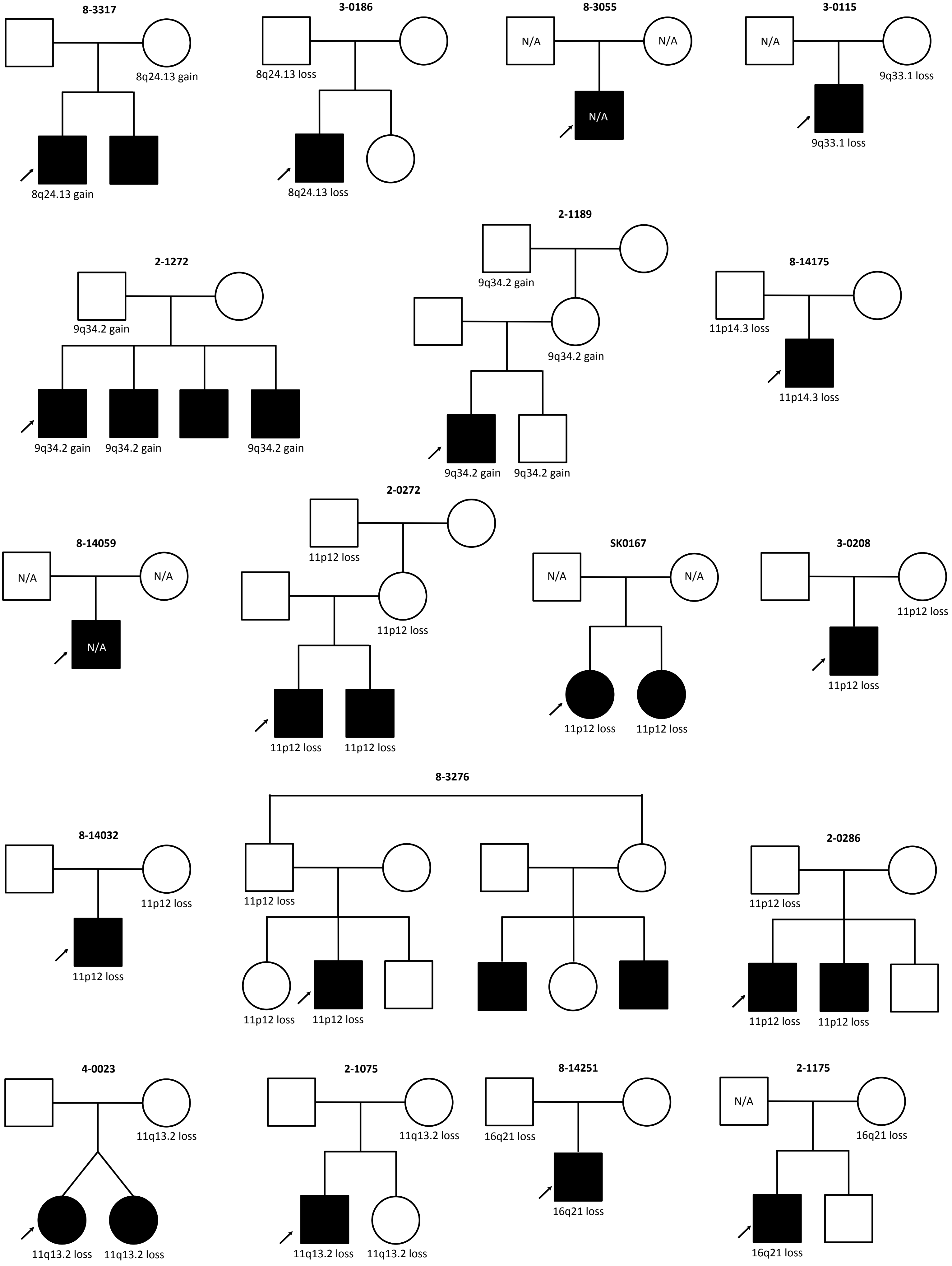

Additional file 2: Pedigree structure for all families in the order in which they are listed in Table 1. In all cases where parental DNA was available for testing, the CNVs described were shown to be inherited. Family SK0167 taken from Marshall *et al.* (American Journal of human Genetics, 2008).
