# Supplementary material for: Identification of candidate intergenic risk loci in autism spectrum disorder
Source: BMC Genomics. 2013 Jul 24;14:499. doi: 10.1186/1471-2164-14-499 (PMC3734099; doi:10.1186/1471-2164-14-499)
Supplement: Additional file 3 — Table of all rare CNVs detected in the individuals described herein. [file 1471-2164-14-499-S3.pdf]

sample	Cytoband	Start	End	size	cnv	Genes
1-0007-003	3q22.1	134,225,395	134,244,869	19,475	gain	*TMEM108*
**1-0007-003**	**3q24**	**146,168,760**	**146,934,953**	**766,194**	**loss**	**-**
1-0007-003	21q21.1	18,179,588	18,188,683	9,096	loss	*NCRNA00157*
**1-0045-004**	**2p16.3**	**51,405,882**	**51,524,684**	**118,803**	**loss**	**-**
1-0045-004	11p15.1	17,083,471	17,118,191	34,721	loss	*PIK3C2A*
**1-0138-004**	**4q13.2**	**66515708**	**66633530**	**117822**	**loss**	**-**
1-0138-004	13q21.31	60567730	60676136	108406	gain	-
**1-0345-005**	**7q21.11**	**80482597**	**80517630**	**35033**	**loss**	**-**
1-0345-005	8q13.3	71701240	71825637	124397	gain	*LACTB2,XKR9*
1-0381-003	2q22.1	137,761,562	137,782,241	20,680	loss	*THSD7B*
**1-0381-003**	**6p21.2**	**40,174,188**	**40,209,324**	**35,137**	**loss**	**-**
1-0381-003	7p21.1	16,804,758	17,712,133	907,376	gain	*AHR,AGR2,AGR3*
1-0381-003	18q21.1	42,536,262	42,662,214	125,953	gain	*PIAS2,ST8SIA5*
**1-0449-003**	**2p16.3**	**52,237,072**	**52,253,660**	**16,589**	**loss**	**-**
1-0455-003	2q34	213,431,545	213,448,326	16,782	loss	-
**1-0455-003**	**4q13.2**	**67,058,506**	**67,075,558**	**17,053**	**loss**	**-**
1-0455-003	4q35.2	190,174,936	190,243,485	68,550	loss	-
1-0455-003	5p13.2	35,058,791	35,113,649	54,859	gain	*PRLR,AGXT2*
1-0455-003	8p12	32,111,587	32,175,992	64,406	loss	*NRG1*
1-0496-003	2p23.3	25,105,894	25,113,024	7,131	loss	*LOC729723*
**1-0496-003**	**2p16.3**	**52,220,120**	**52,238,172**	**18,053**	**gain**	**-**
1-0496-003	ChrX	1	154,913,754	154,913,754	Complex	*46, XXY*
**2-0082-004**	**4q13.2**	**67,045,815**	**67,134,170**	**88,356**	**loss**	**-**
**2-0139-003**	**6p21.2**	**40023327**	**40062155**	**38828**	**gain**	**-**
2-0272-003	7q36.2	153742175	153788452	46277	loss	*DPP6*
**2-0272-003**	**11p12**	**40379668**	**40550356**	**170688**	**loss**	**-**
**2-0286-003**	**11p12**	**42243624**	**42279094**	**35470**	**loss**	**-**
2-1075-003	1q32.1	202,359,648	202,376,233	16,586	loss	*SOX13,ETNK2*
**2-1075-003**	**11q13.2**	**68,486,121**	**68,500,238**	**14,118**	**loss**	**-**
2-1075-003	11q22.3	102,443,261	102,710,521	267,261	gain	*DYNC2H1,DCUN1D5*
2-1175-003	8q23.1	110,133,336	110,249,651	116,316	gain	*TRHR*
2-1175-003	12p13.33	424,352	485,647	61,296	loss	*B4GALNT3*
**2-1175-003**	**16q21**	**61,658,675**	**61,755,232**	**96,558**	**loss**	**-**
**2-1189-003**	**9q34.2**	**136480334**	**136598491**	**118157**	**gain**	**-**
2-1189-003	21q21.3	26100421	26154069	53648	loss	-
**2-1213-003**	**3p22.3**	**34,984,049**	**35,102,773**	**118,725**	**loss**	**-**

2-1213-003	7p11.2	54,174,542	54,198,069	23,528	gain	-
2-1272-003	9q34.3,9q34.2	136,479,329	136,604,233	124,905	gain	-
**2-1368-003**	**6p21.2**	**40,174,188**	**40,210,694**	**36,507**	**loss**	**-**
2-1368-003	20p13	1,541,849	1,817,073	275,225	gain	*SIRPG,SIRPB1*
3-0044-000	4q12	55608691	55681319	72628	loss	*KDR*
**3-0044-000**	**8p12**	**34923482**	**34956067**	**32585**	**loss**	**-**
**3-0100-000**	**3p22.3**	**35,086,691**	**35,094,736**	**8,046**	**gain**	**-**
3-0100-000	Xp11.3	44,805,180	45,218,177	412,998	gain	*CXorf36,KDM6A*
3-0115-000	8q24.13	125,558,088	125,576,651	18,564	loss	*RNF139,TATDN1*
**3-0115-000**	**9q33.1**	**119,314,967**	**119,319,559**	**4,593**	**loss**	**-**
3-0139-000	2p22.3	33,636,585	33,668,888	32,304	loss	*RASGRP3,FAM98A*
**3-0139-000**	**6p21.2**	**40,021,898**	**40,078,515**	**56,618**	**gain**	**-**
**3-0186-000**	**8q24.13**	**123583028**	**123639417**	**56389**	**loss**	**-**
**3-0208-000**	**11p12**	**40,468,058**	**40,492,541**	**24,484**	**loss**	**-**
3-0208-000	22q12.1	27,732,539	27,742,209	9,671	gain	*ZNRF3*
**3-0300-000**	**8p12**	**34,925,149**	**34,957,854**	**32,706**	**loss**	**-**
4-0023-003	5p13.1	40,964,760	40,979,607	14,848	loss	*C7*
**4-0023-003**	**11q13.2**	**68,486,121**	**68,493,638**	**7,518**	**loss**	**-**
8-3055-004	1p21.1	105122765	105198703	75938	gain	-
**8-3055-004**	**9q33.1**	**119254497**	**119374796**	**120299**	**loss**	**-**
**8-3093-004**	**3q24**	**146575437**	**146631141**	**55704**	**loss**	**-**
8-3093-004	8q11.23	53518178	53976643	458465	gain	*RB1CC1,FAM150A*
8-3093-004	9p23	9821068	9935648	114580	loss	*PTPRD*
8-3093-004	10q22.3	79736155	79921727	185572	gain	-
**8-3276-003**	**11p12**	**42243624**	**42279094**	**35470**	**loss**	**-**
8-3276-003	19p13.3	423980	483066	59086	gain	*CDC34,C19orf20,ODF3L2,MADCAM1*
**8-6258-3**	**7q21.11**	**80431202**	**80512022**	**80820**	**loss**	**-**
8-14032-600	3p11.2	87266573	87301699	35126	gain	-
8-14032-600	9p23	9698107	9746868	48761	loss	*PTPRD*
**8-14032-600**	**11p12**	**41990280**	**42021250**	**30970**	**loss**	**-**
8-14032-600	Xp21.1	35770411	35851450	81039	gain	*CXorf22*
8-14059-1020	6q16.1	95704450	95735253	30803	gain	-
8-14059-1020	9q31.1	103977934	104019906	41972	loss	-
**8-14059-1020**	**11p14.3**	**24262511**	**24303132**	**40621**	**loss**	**-**
8-14144-2420	2p22.2	36487324	36559251	71927	loss	*CRIM1*
**8-14144-2420**	**2p16.3**	**51157414**	**51225851**	**68437**	**loss**	**-**
8-14144-2420	5q21.1	101133897	101394315	260418	loss	-

8-14144-2420	15q25.3	83596208	83910586	314378	gain	*AKAP13*
8-14144-2420	15q26.1	88639397	88800132	160735	gain	*ZNF774,IQGAP1*
8-14175-2820	3q28	191861309	192078454	217145	gain	*SNAR-I*
**8-14175-2820**	**11p14.3**	**24177612**	**24316053**	**138441**	**loss**	**-**
8-14175-2820	13q12.11	19256616	19323948	67332	gain	*ZMYM5*
8-14181-2940	6q22.33	127962618	127988891	26273	gain	-
**8-14181-2940**	**8p12**	**34923482**	**34956067**	**32585**	**loss**	**-**
8-14181-2940	15q21.1	46054818	46111620	56802	loss	-
8-14181-2940	15q25.2	82906265	82985247	78982	gain	*FLJ43276,SCAND2,ZSCAN2*
8-14186-3050	3p14.2	60013672	60200705	187033	loss	*FHIT*
8-14186-3050	4q12	53710998	53795761	84763	loss	*SCFD2*
**8-14186-3050**	**4q13.2**	**66515708**	**66633530**	**117822**	**loss**	**-**
8-14186-3050	4q13.3	71197092	71316473	119381	loss	*PROL1,C4orf35,SMR3B,SMR3A*
8-14186-3050	10q26.3	133210184	133693052	482868	gain	*PPP2R2D,BNIP3*
8-14186-3050	17p11.2	20373349	20540089	166740	loss	-
**8-14208-3350**	**4q13.2**	**66505324**	**66633530**	**128206**	**loss**	**-**
8-14208-3350	6p24.3	7076523	7622856	546333	gain	*RIOK1,SNRNP48,DSP,SSR1,CAGE1,RREB1*
**8-14208-3350**	**11p14.3**	**24262511**	**24303132**	**40621**	**loss**	**-**
**8-14208-3350**	**11p12**	**40304880**	**40703298**	**398418**	**gain**	**-**
8-14208-3350	11q24.2	123700734	123771013	70279	loss	*OR8B2*
8-14208-3350	17p11.2	21480206	21654390	174184	loss	-
8-14243-3670	5q21.2	103517908	103561783	43875	loss	-
**8-14243-3670**	**8p12**	**34923482**	**34956067**	**32585**	**loss**	**-**
8-14251-3750	4q28.1	125500302	125544407	44105	loss	-
**8-14251-3750**	**16q21**	**61650435**	**61787984**	**137549**	**loss**	**-**
**8-3317-003**	**8q24.13**	**123572785**	**123625681**	**52896**	**gain**	**-**
**8-3394-003**	**2p16.3**	**51157414**	**51189362**	**31948**	**loss**	**-**
**8-3394-003**	**2p16.3**	**51439897**	**51479683**	**39786**	**loss**	**-**
8-3394-003	15q26.3	99845991	99968896	122905	gain	*PCSK6*
8-3394-003	15q26.3	100041294	100105994	64700	gain	*TARSL2*

Additional file 3: Table detailing all rare CNVs detected in the samples described. Intergenic CNVs shown in Table 1 are shown in bold.
